# Gait stability in peripheral artery disease: a phase-dependent analysis using gait tube methodology

**DOI:** 10.3389/fresc.2026.1725201

**Published:** 2026-02-12

**Authors:** Arash Mohammadzadeh Gonabadi, Iraklis I. Pipinos, Sara A. Myers, Farahnaz Fallahtafti

**Affiliations:** 1 Research Institute, Madonna Rehabilitation Hospitals, Omaha, NE, United States; 2 Department of Biomechanics, University of Nebraska at Omaha, Omaha, NE, United States; 3 Department of Surgery, University of Nebraska Medical Center, Omaha, NE, United States; 4 Department of Surgery and Research Service, Nebraska-Western Iowa Veterans Affairs Medical Center, Omaha, NE, United States

**Keywords:** fall risk, gait tube stability, peripheral artery disease (PAD), phase-dependent analysis, walking stability

## Abstract

**Introduction:**

Peripheral Artery Disease (PAD) affects over eight million U.S. adults, impairing mobility, quality of life, and increasing fall and cardiovascular risks. PAD reduces lower limb blood flow and contributes to neuromuscular dysfunction, leading to unstable gait. Traditional stability metrics often miss phase-specific changes. Gait Tube Stability (GTS) is a phase-dependent, three-dimensional method that analyzes center of mass (COM) velocity using ellipsoidal variability to detect directional instability. This study examined whether GTS can identify phase-specific gait deficits in PAD compared with age-matched controls.

**Methods:**

Fifty-two PAD patients and 132 healthy individuals walked on a force-instrumented treadmill at self-selected speeds. GTS metrics-including ellipsoid volume and direction-specific variabilities (AP, ML, VT)-were computed from 3D COM velocity. Data were segmented by gait phase and analyzed using Wilcoxon rank-sum tests and Statistical Parametric Mapping (SPM).

**Results:**

PAD patients exhibited significantly lower ellipsoid volumes (3.07 × 10^5^ mm³/s³, *p* < 0.001) and reduced VT variability (40.29 mm/s, *p* < 0.001) compared to controls (1.02 × 10^6^ mm³/s³ and 93.60 mm/s). No significant differences were found in AP (*p* = 0.1062) or ML (*p* = 0.6467) variability. Correlation between ellipsoid volume and total variability was weak in PAD (*r* = 0.06, *p* = 0.6587) but moderate in controls (*r* = 0.55, *p* < 0.001), indicating impaired multidirectional coordination in PAD. Correlation analysis revealed a significant association between ellipsoid volume and total variability in controls but not in PAD, and Fisher's *r*-to-*z* test confirmed a significant between-group difference (*p* = 0.005).

**Discussion:**

GTS revealed phase-specific gait deficits in PAD, especially during weight acceptance and early stance, indicating a constrained, energy-conserving strategy that may elevate fall risk. By detecting critical instability phases, GTS can guide targeted physical therapy, assistive device use, and optimal timing for robotic or exoskeleton support-supporting personalized interventions and offering a sensitive tool for clinical gait stability assessment.

## 1 Introduction

Peripheral artery disease (PAD) affects more than 200 million adults globally, including approximately 8.5 million in the United States. Its prevalence reaches 20% among individuals aged 70 or older (1–3). The underlying problem is progressive atherosclerotic plaque that narrows the arteries supplying the legs, limits blood flow, and starves working muscles of oxygen (1–3). This imbalance between oxygen supply and demand leads to muscle ischemia during walking, causing debilitating leg pain that forces the person with PAD to stop walking (4, 5). Therefore, PAD contributes to significant declines in physical function, including reduced daily activity levels, lower self-reported mobility, and diminished quality of life (6, 7). Among these impairments, gait instability due to muscular dysfunction and the associated elevated fall risk remain critical yet understudied consequences with substantial implications for patient safety, autonomy, and rehabilitation success.

PAD pathophysiology extends beyond vascular insufficiency to include skeletal muscle myopathy and sensory-motor nerve dysfunction in the lower limbs, increasing susceptibility to gait instability and falls (8). Previous studies have demonstrated that vulnerability to balance loss and external perturbations during walking is phase-dependent (9–12). Weight acceptance and early single-limb support are considered particularly critical phases, as rapid load transfer and reduced base of support place high demands on neuromuscular control (9–12). Perturbations applied during these stance-related phases have been shown to elicit larger destabilizing responses and require greater corrective actions than those applied during swing (9–12). These findings highlight the importance of phase-specific stability assessment when evaluating fall risk and gait control, particularly in populations with impaired sensorimotor function, such as individuals with PAD. Computerized Dynamic Posturography has shown significant postural control deficits in PAD, especially under vestibular and somatosensory challenges (8, 13). The Sensory Organization Test indicates increased reliance on hip strategies and reduced vestibular input, while the Motor Control Test reveals delayed response latencies and diminished amplitude scaling—both signs of impaired neuromuscular function (13). These deficits, along with altered center-of-gravity alignment and lower equilibrium scores, suggest a reduced ability to recover from postural disturbances (14). Gardner and Montgomery reported that PAD patients had 28% shorter single-leg stance time, 86% more frequent stumbling, and a 73% higher fall rate than controls—associations strongly linked to reduced walking capacity and lower physical activity levels (14). Previous studies have shown that individuals with PAD rely more heavily on hip strategies during standing balance tasks (8, 14), suggesting compensatory proximal control in response to impaired distal strength and sensory feedback. Such reliance on hip-dominant control may extend to walking, where increased or altered hip motion could influence center-of-mass regulation, particularly in the mediolateral and anteroposterior directions. These adaptations may lead to changes in inter-directional coordination of COM velocity across the gait cycle, especially during stance-related phases when balance demands are highest (10, 15, 16). The Gait Tube Stability (GTS) framework is well-suited to capture these multidirectional and phase-dependent coordination patterns during walking (17, 18). Individuals with peripheral artery disease typically adopt slower self-selected walking speeds as a strategy to delay pain onset and reduce metabolic demand associated with intermittent claudication (13). Reduced walking speed influences spatiotemporal gait parameters and can affect center-of-mass velocity and its variability (14). Therefore, alterations in gait stability metrics observed in PAD may reflect both disease-specific neuromuscular impairments and speed-related adaptations to pain and discomfort (8, 13). Intermittent claudication is a hallmark symptom of peripheral artery disease, characterized by exercise-induced pain, cramping, or discomfort in the lower extremity muscles that occurs during walking due to inadequate arterial blood flow (5, 8, 14). These symptoms typically subside with rest as metabolic demand decreases and perfusion is partially restored. Intermittent claudication limits walking capacity, alters gait mechanics, and contributes to functional impairment and increased fall risk in individuals with PAD.

Traditional biomechanical measures of walking stability, such as the margin of stability (MoS), quantify the dynamic interaction between the center of mass (COM) and the base of support (10, 19). In the anterior-posterior (AP) direction, MoS values are typically negative, reflecting the fall-forward condition required for locomotion, with more negative values indicating reduced stability and a greater urgency to place the next footstep quickly to maintain balance (15, 17–19). In the mediolateral direction, MoS is usually positive, except during unstable phases when active compensatory movements are required to avoid falls (15). While MoS has provided valuable insights into stability at discrete gait events (e.g., heel contact), it cannot identify the phase-specific variations in stability across the entire gait cycle (19). While discrete stability measures such as the margin of stability are informative at specific gait events (e.g., heel strike), they do not capture how instability evolves continuously throughout the gait cycle (10, 19). This limitation is particularly relevant for individuals with peripheral artery disease, in whom ischemia-related muscle weakness, altered sensory feedback, and fatigue progressively affect gait control during stance (20–23). Prior studies have reported PAD-related alterations in joint mechanics, muscle activation, and propulsion that emerge during weight acceptance, mid-stance, and push-off rather than exclusively at discrete events (21, 24, 25). Consequently, instability-related adaptations in PAD may occur between discrete gait events and remain undetected by event-based metrics alone (15). Continuous, phase-dependent approaches such as Gait Tube Stability are therefore necessary to characterize when and how gait instability develops across the gait cycle in this population (10, 16).

Several established approaches have been used to quantify gait stability and locomotor control, each capturing distinct characteristics of movement dynamics. Lyapunov exponent–based measures assess local sensitivity to small perturbations by quantifying the divergence of nearby trajectories in state space, thereby reflecting the system's resistance to infinitesimal disturbances (15, 19, 26–29). Entropy-based metrics, such as approximate and sample entropy, characterize the regularity and complexity of gait time series and provide insight into the predictability of neuromotor control (30–32). While these approaches have proven valuable in identifying altered locomotor dynamics in pathological populations, they do not explicitly resolve how stability evolves continuously across the gait cycle or how control differs across anatomical directions (17, 18). Gait Tube Stability addresses this gap by quantifying continuous, phase-dependent variability of center-of-mass velocity in the mediolateral, anteroposterior, and vertical directions (17, 18). By integrating multidirectional COM behavior throughout the gait cycle, GTS enables identification of when instability emerges and how coordination strategies differ across phases, providing complementary information to Lyapunov- and entropy-based measures rather than replacing them (17, 18).

To overcome the limitations of traditional discrete-event stability metrics, Gonabadi et al. introduced the Gait Tube Stability (GTS) method—a continuous, three-dimensional, and phase-dependent approach for assessing gait stability across the full gait cycle (17, 18). This method was first introduced by a presentation by Seongwoo Mun and Nathaniel Hunt at the 2025 Human Movement Variability Conference, in which they introduced an approach to quantify recovery time following perturbations during human walking (18). This study applied the GTS framework to compare gait stability between PAD patients and age-matched controls. We hypothesized that PAD would be associated with smaller ellipsoid volumes, reduced VT variability, and weaker coordination across directions—reflecting impaired sensorimotor integration. These phase-specific deficits likely represent compensatory strategies due to vascular and neuromuscular dysfunction and may contribute to increased fall risk. Ellipsoid volume is used as an integrated measure of multidirectional center-of-mass velocity variability. We also hypothesized that ellipsoid volume would relate to traditional variability metrics by capturing the combined dispersion of COM velocity across anatomical directions, while also allowing assessment of phase-dependent stability patterns. Identifying such instabilities can guide targeted rehabilitation to promote safer walking and improve quality of life in individuals with PAD.

## 2 Methods

### 2.1 Participant

The study population included 132 healthy individuals (age: 64.92 ± 9.68 years; height: 173.79 ± 7.58 cm; weight: 83.75 ± 18.42 kg) and 52 individuals with PAD (age: 64.32 ± 6.54 years; height: 175.60 ± 7.52 cm; weight: 88.85 ± 16.92 kg). All PAD participants were recruited from the Vascular Surgery Department at the Nebraska and Western Iowa Veterans Affairs Medical Center (VAMC) in Omaha, Nebraska. Eligibility was determined by a board-certified vascular surgeon based on a documented history of chronic claudication, exercise-limiting leg pain confirmed through clinical evaluation and a standardized walking test, and an ankle-brachial index below 0.90 at rest (33). Additional inclusion criteria required stable medication regimens for managing hypertension, dyslipidemia, and diabetes, along with stable control of associated risk factors for at least six weeks before participation. Exclusion criteria included rest pain or tissue loss consistent with Fontaine stages III or IV (34, 35), recent acute ischemic events (36), thromboembolic disease (37), traumatic injuries, or any other condition that limited walking ability independent of PAD. All participants provided written informed consent, and the study protocol received approval from the Institutional Review Board of the VAMC and the University of Nebraska, in accordance with the ethical principles of the Declaration of Helsinki. The study used the ethical principles outlined in the Declaration of Helsinki.

### 2.2 Data collection

All participants wore form-fitting motion capture suits and standardized footwear. Participant recruitment and data collection were conducted during a single, continuous study phase. Individuals with PAD were recruited through the Vascular Surgery Department at the Nebraska–Western Iowa Veterans Affairs Medical Center, while age-matched healthy control participants were recruited from the local community. Recruitment occurred on a rolling basis until the target sample size was achieved, with no multiple recruitment waves or intervention phases. Following enrollment and informed consent, all participants completed one laboratory visit in which gait data were collected during treadmill walking. For participants with PAD, data collection was performed prior to the onset of claudication pain to ensure that gait characteristics reflected baseline walking behavior rather than pain-induced compensatory strategies. The overall study timeline and data collection workflow have been clarified here to improve methodological transparency. Participants completed a treadmill familiarization session prior to data collection to ensure comfortable and steady walking. Following familiarization, each participant walked at a self-selected preferred speed to reflect natural gait behavior. The average preferred walking speed was 0.68 ± 0.47 m/s for the control group and 0.56 ± 0.45 m/s for the PAD group. All gait data were time-normalized to the gait cycle, and stability analyses focused on phase-dependent variability of center-of-mass velocity rather than absolute speed-dependent magnitudes. This approach minimizes the influence of between-group differences in speed while preserving physiologically relevant gait control characteristics. Thirty-three retroreflective markers were placed on lower-extremity landmarks, and 3D kinematic data were collected using a 12-camera, 60 Hz motion capture system (Motion Analysis Corp., CA, USA). Participants walked for two minutes on a force-instrumented treadmill (AMTI, MA, USA) at the University of Nebraska at Omaha. At least 90 consecutive gait cycles were recorded per subject during steady-state walking. For PAD participants, data were analyzed only before claudication pain onset. The treadmill incline was set to 0% to minimize confounding. The protocol was approved by IRBs at the University of Nebraska Medical Center and the Nebraska-Western Iowa VA Medical Center (IRB#1576204), and written informed consent was obtained from all participants.

### 2.3 Data analysis

**Figure 1:**
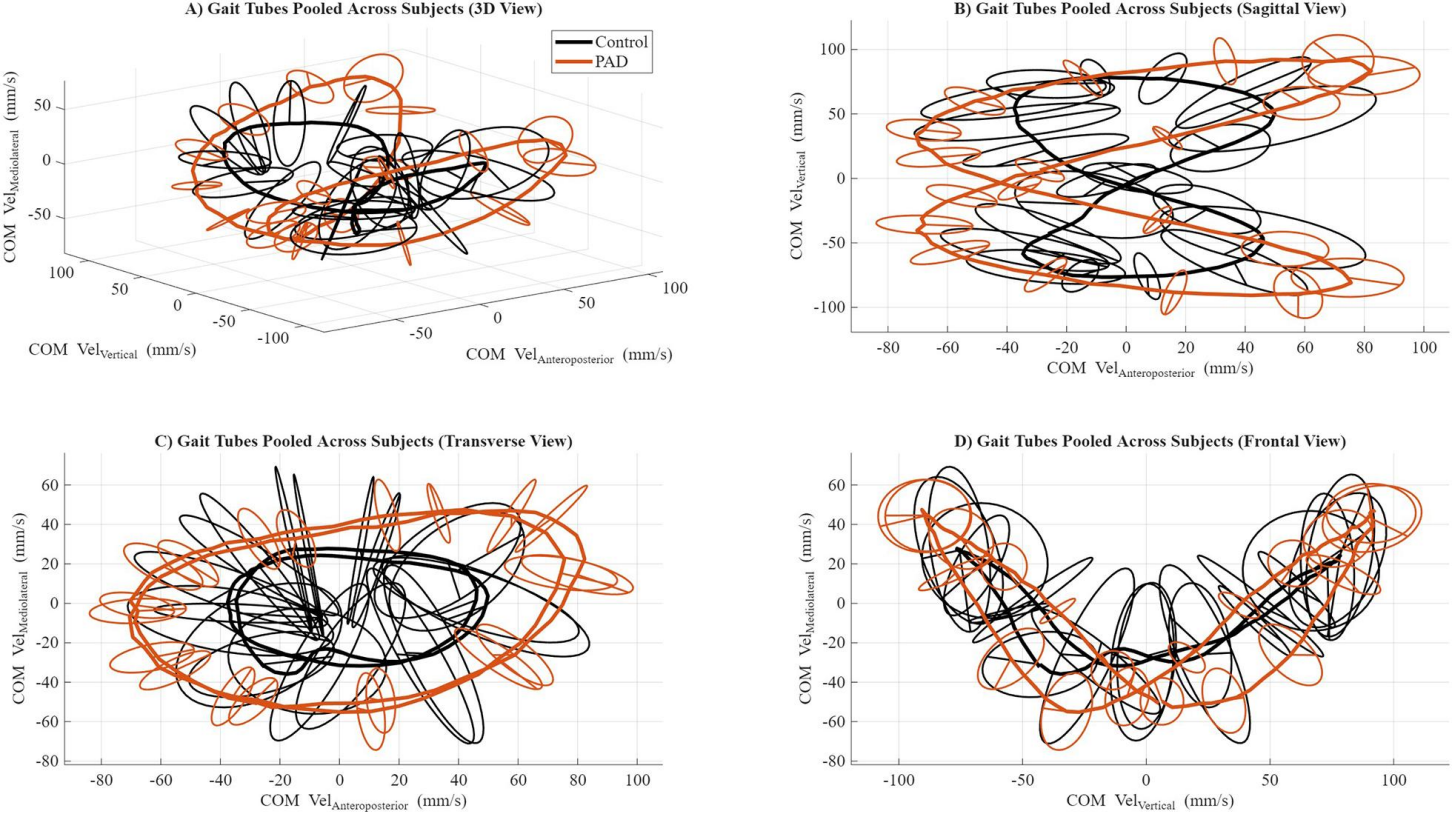
Gait tubes were pooled across subjects under each group. COM velocity trajectories were first time-normalized to the gait cycle for each participant. Group-level gait tubes were generated by averaging the normalized trajectories across participants within each group at each gait-cycle percentage, yielding grand-mean representations for the control and PAD groups. **(A)** Three-dimensional view of the center of mass (COM) velocity trajectories in velocity state space. **(B)** Sagittal view showing the COM's vertical and anteroposterior components. **(C)** The transverse view shows the COM mediolateral vs. anteroposterior components. **(D)** The frontal view shows the COM mediolateral vs. vertical components. Each color corresponds to a participant group: the control group is represented by a black line, and the PAD group is shown in red. Ellipsoids were computed at each gait phase and plotted to represent local variability in COM velocity across strides.

No *a priori* sample size or power analysis was performed for the present study. The dataset analyzed here was drawn from an existing cohort originally collected to examine gait characteristics in individuals with peripheral arterial disease and age-matched controls. The current investigation is a secondary, exploratory analysis aimed at evaluating the sensitivity of the Gait Tube Stability framework to detect phase-dependent differences in center-of-mass velocity variability. Despite the absence of an *a priori* power calculation, statistically significant between-group differences were identified using nonparametric and Statistical Parametric Mapping–based (SPM) analyses (38, 39), indicating that the available sample size was sufficient to detect meaningful effects within the scope of the present study. The COM position was estimated using pelvis kinematics (19, 40). Specifically, the COM position was calculated as the average position of pelvis markers (19, 40), including the sacrum, bilateral anterior superior iliac spines (ASIS), and bilateral posterior superior iliac spines (PSIS). This pelvis-based approximation was used to capture the body's COM motion during walking (19, 40). Gait cycles were identified using ground reaction force data (19, 40, 41). Heel-strike events were detected from the vertical GRF signal, and each gait cycle was defined from heel strike to the subsequent heel strike of the same limb (41). All gait cycles were time-normalized to 0%–100% prior to further analysis to enable phase-dependent comparisons across participants and groups. GTS quantifies stride-to-stride variability in COM velocity across anteroposterior (AP), mediolateral (ML), and vertical (VT) directions by constructing a 3 × 3 covariance matrix at each normalized time point (17, 18). Direction-specific ellipsoids are derived from this matrix, with their volumes serving as localized measures of dynamic variability (17, 18). These ellipsoids are projected onto Frenet–Serret planes to retain anatomical context and smoothed temporally to create a continuous “gait tube” visualization of COM variability (17, 18). For visualization and group-level comparison, COM velocity trajectories were pooled across participants within each group following time normalization. For each participant, COM velocity trajectories were segmented from heel strike to the subsequent heel strike of the same limb and normalized to 0%–100% of the gait cycle. Group-level trajectories were then obtained by computing the point-wise mean across participants at each percentage of the gait cycle for each velocity direction. Gait tubes and ellipsoid representations were constructed from these group-averaged trajectories, representing the grand mean behavior of each group rather than concatenated individual trials (17). This approach reveals phase-specific instability patterns often missed by traditional methods. Prior studies showed GTS sensitivity to perturbations from hip exoskeleton torque, especially during early-to-mid stance—critical for postural control (17). Ellipsoid volumes also correlate strongly with total variability, supporting their use as robust indicators of gait stability (17). GTS thus offers valuable insights for clinical assessment and assistive technology development. To compare GTS metrics between the Control and PAD groups, statistical testing was conducted across the gait cycle for each dependent variable, including ellipsoid volume and direction-specific linear variabilities (AP, ML, VT) (17, 42–44). Between-group comparisons of gait tube stability metrics and directional variability measures were performed using the Wilcoxon rank-sum test (Mann–Whitney U test) (17, 42–46), which is appropriate for independent samples with unequal group sizes. This nonparametric approach was selected because several outcome measures exhibited non-normal distributions. In addition, SPM (38, 39) was used to compare continuous gait-cycle waveforms between the control and PAD groups while appropriately controlling for multiple comparisons across the entire 0%–100% gait cycle. Within-group associations between ellipsoid volume and total variability were assessed using Pearson correlation coefficients. To formally compare whether the strength of these relationships differed between the control and PAD groups, Fisher's *r*-to-*z* transformation (16, 47, 48) was applied to the correlation coefficients obtained for each group. This approach enables direct statistical comparison of independent correlation coefficients and was used to test for group differences in coordination between multidirectional COM variability measures. Each group's Pearson correlation coefficients were calculated between ellipsoid volume and total linear variability to assess three-dimensional stability coordination. All analyses were performed using MATLAB R2024b (MathWorks, Natick, MA, USA), ensuring reproducibility and alignment with previously published GTS methods. All statistical significances were assessed at an alpha level of 0.05. For visualization and group-level analysis, gait tube trajectories were constructed using pooled time-normalized data (17, 18). Individual gait cycles were first time-normalized to 0%–100% of the gait cycle and averaged across participants (17, 18). These participant-level mean trajectories were then averaged across all participants within each group to generate grand-mean group trajectories shown in Figure 1 (17, 18).

## 3 Results

GTS analysis revealed significant differences in gait stability between groups. Pooled 3D COM velocity trajectories (Figure 1A) show distinct patterns for Control and PAD participants. The sagittal view (Figure 1B) highlights reduced VT excursion and constrained AP movement in PAD, while the transverse (Figure 1C) and frontal (Figure 1D) views illustrate ML–AP and ML–VT relationships with ellipsoids representing phase-specific variability. Although the mean COM velocity trajectories of the control and PAD groups partially overlap when projected onto individual planes, the GTS framework quantifies stability based on the volume and radial dispersion of the gait tube rather than the mean trajectory location. Visual inspection of Figure 1 demonstrates that the control group exhibits a thicker tube with larger surrounding rings, reflecting greater multidirectional COM velocity variability. In contrast, the PAD group shows a thinner tube with reduced radial dispersion, corresponding to a smaller ellipsoid volume and more constrained COM control. These differences are not captured by examining trajectory excursions alone but emerge from the volumetric representation central to the GTS approach.

**Figure 2:**
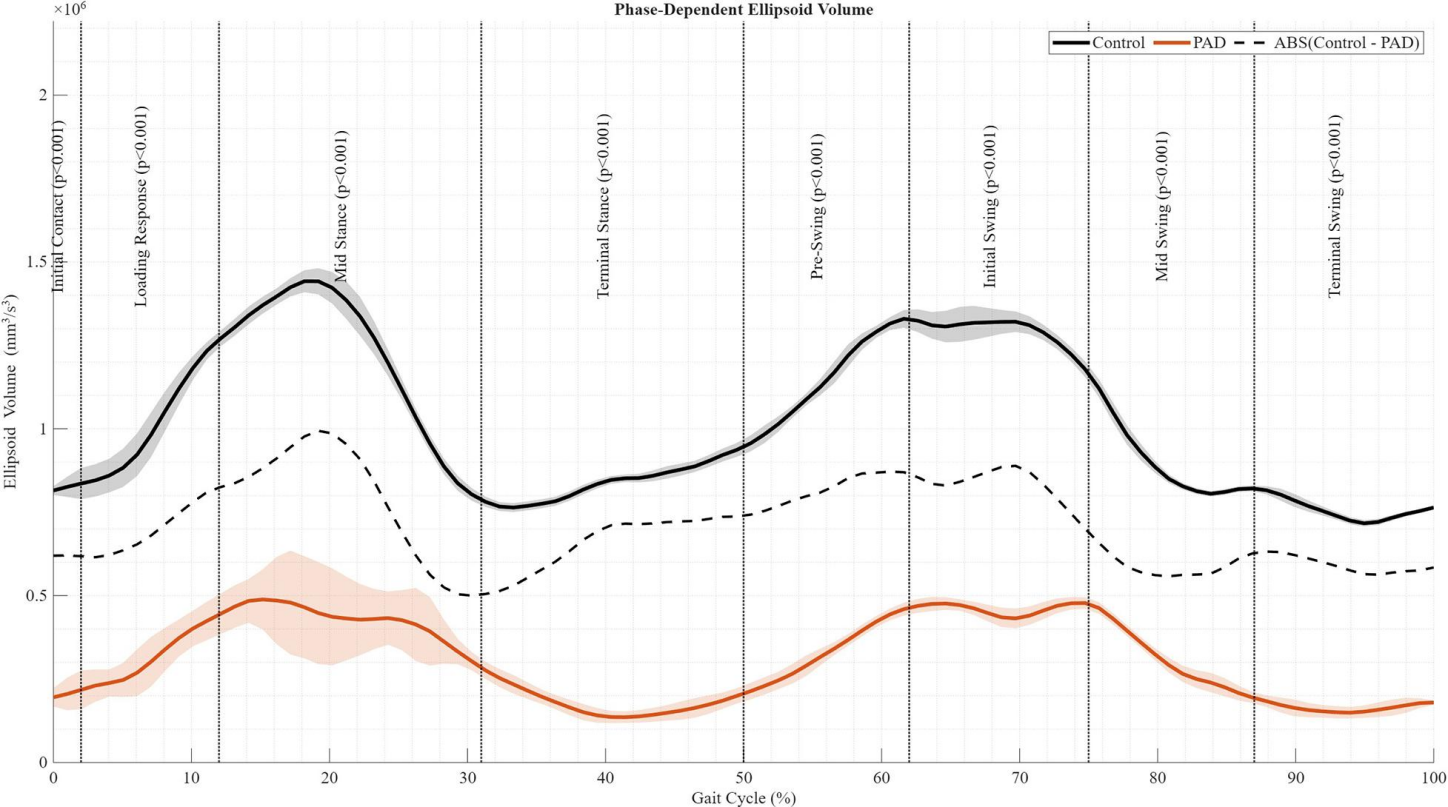
Phase-dependent metrics and supporting signals across walking conditions for each group. Phase-dependent ellipsoid volumes across the gait cycle for all walking conditions. Solid lines show the mean ellipsoid volume for the control and PAD groups. The dashed line represents the absolute phase-dependent difference between groups (Control—PAD). Based on the Statistical Parametric Mapping (SPM), statistically significant differences from the Control group are indicated by the corresponding *p*-values in each gait-phase definition. The shaded band is the standard deviation.

Figure 2 presents the phase-dependent ellipsoid volume for the control and PAD groups, along with a third curve representing the absolute between-group difference, defined as Control minus PAD. Positive values across the gait cycle indicate larger ellipsoid volumes in the control group. Phase-dependent between-group differences were evaluated using SPM applied to the time-normalized gait-cycle waveforms, with significance assessed separately for each clinically defined gait phase. Significant between-group differences were identified across all gait phases, including Initial Contact (0%–2%, *p* < 0.001), Loading Response (2%–12%, *p* < 0.001), Mid Stance (12%–31%, *p* < 0.001), Terminal Stance (31%–50%, *p* < 0.001), Pre-Swing (50%–62%, *p* < 0.001), Initial Swing (62%–75%, *p* < 0.001), Mid Swing (75%–87%, *p* < 0.001), and Terminal Swing (87%–100%, *p* < 0.001). These findings indicate pervasive phase-dependent differences in gait tube stability between the control and PAD groups across the entire gait cycle. These results demonstrate that PAD-related alterations in center-of-mass variability are phase-specific and not uniformly distributed across the gait cycle.

**Figure 3:**
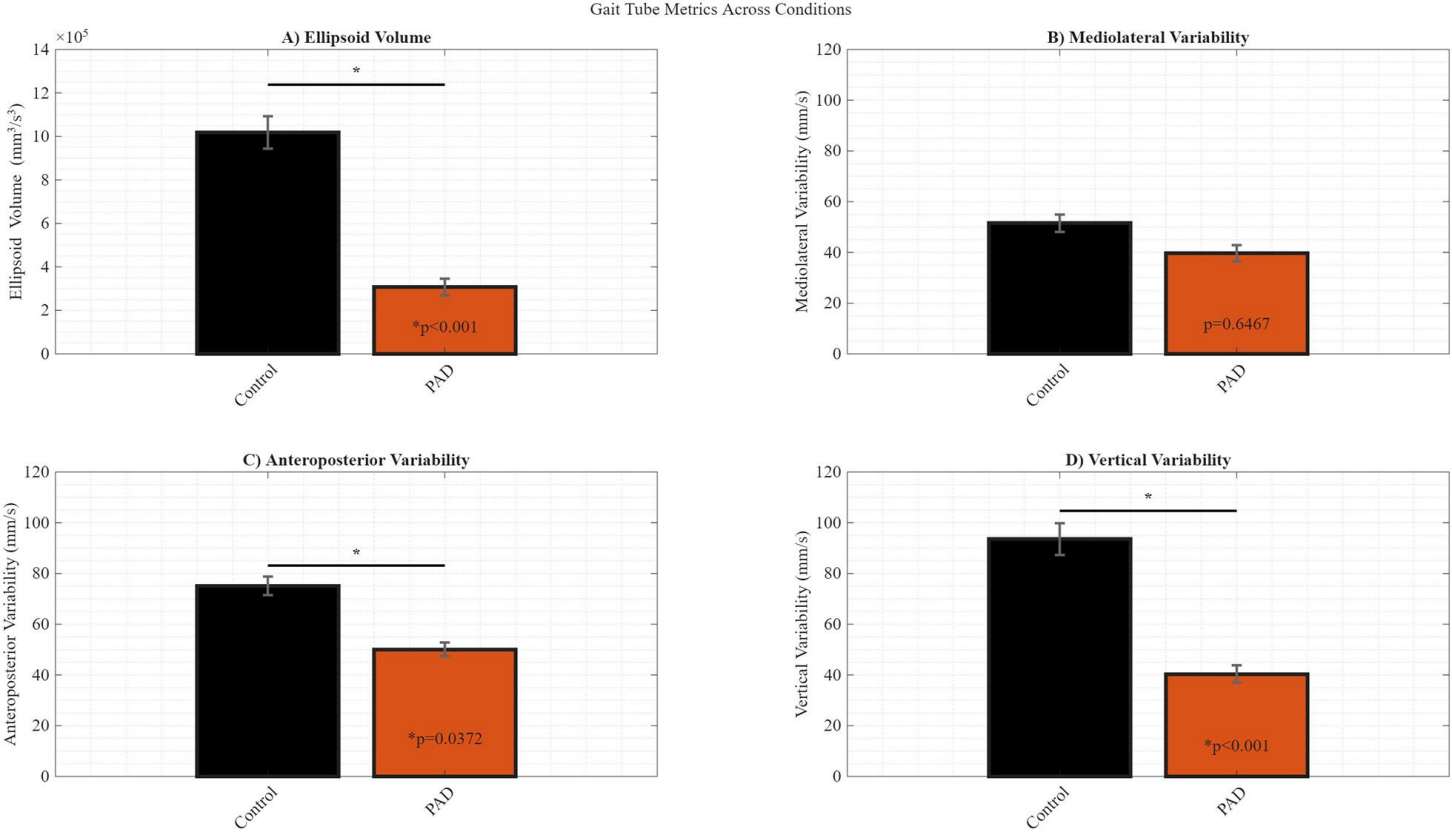
Summary of gait tube metrics averaged across the gait cycle for each group. **(A)** Mean ellipsoid volume. **(B)** Mediolateral linear variability. **(C)** Anteroposterior linear variability. **(D)** Vertical linear variability. Bars represent condition-wise means across subjects, and error bars indicate standard deviations. Statistical significance is determined using the Wilcoxon rank-sum test relative to the control group. If a statistically significant difference was found between the PAD and control groups, the corresponding *p*-values are reported within the PAD bar graphs.

Phase-dependent ellipsoid volumes across the gait cycle (Figure 2) show significantly reduced variability in the PAD group, with the most prominent difference during weight acceptance (*p* < 0.001), marking a critical phase of reduced stability. Averaged gait tube metrics (Figure 3) further illustrate group differences. PAD participants had a significantly lower mean ellipsoid volume (3.07 × 10^5^ mm^3^/s^3^) compared to controls (1.02 × 10^6^ mm^3^/s^3^; *p* = 0.0003). ML variability (Figure 3B) showed no significant difference (PAD: 39.70 mm/s; Control: 51.58 mm/s; *p* = 0.6467); however, AP variability (Figure 3C; PAD: 49.99 mm/s; Control: 75.07 mm/s; *p* = 0.0372) showed a significant difference. In addition, VT variability (Figure 3D) was significantly lower in PAD (40.29 mm/s) than in controls (93.60 mm/s; *p* < 0.001), suggesting a deliberate reduction in vertical motion—likely a compensatory strategy to enhance balance. Ellipsoid volume differed significantly between groups (*p* < 0.001). Direction-specific analyses revealed that this difference was primarily driven by the vertical component of COM velocity variability (*p* < 0.001), with a smaller but significant contribution from the anteroposterior component (*p* = 0.037). Mediolateral variability did not differ significantly between groups (*p* = 0.647). Although ellipsoid volume represents an integrated measure of multidirectional COM variability, these findings indicate that vertical—and to a lesser extent anteroposterior—control predominantly underlies the observed group differences.

**Figure 4:**
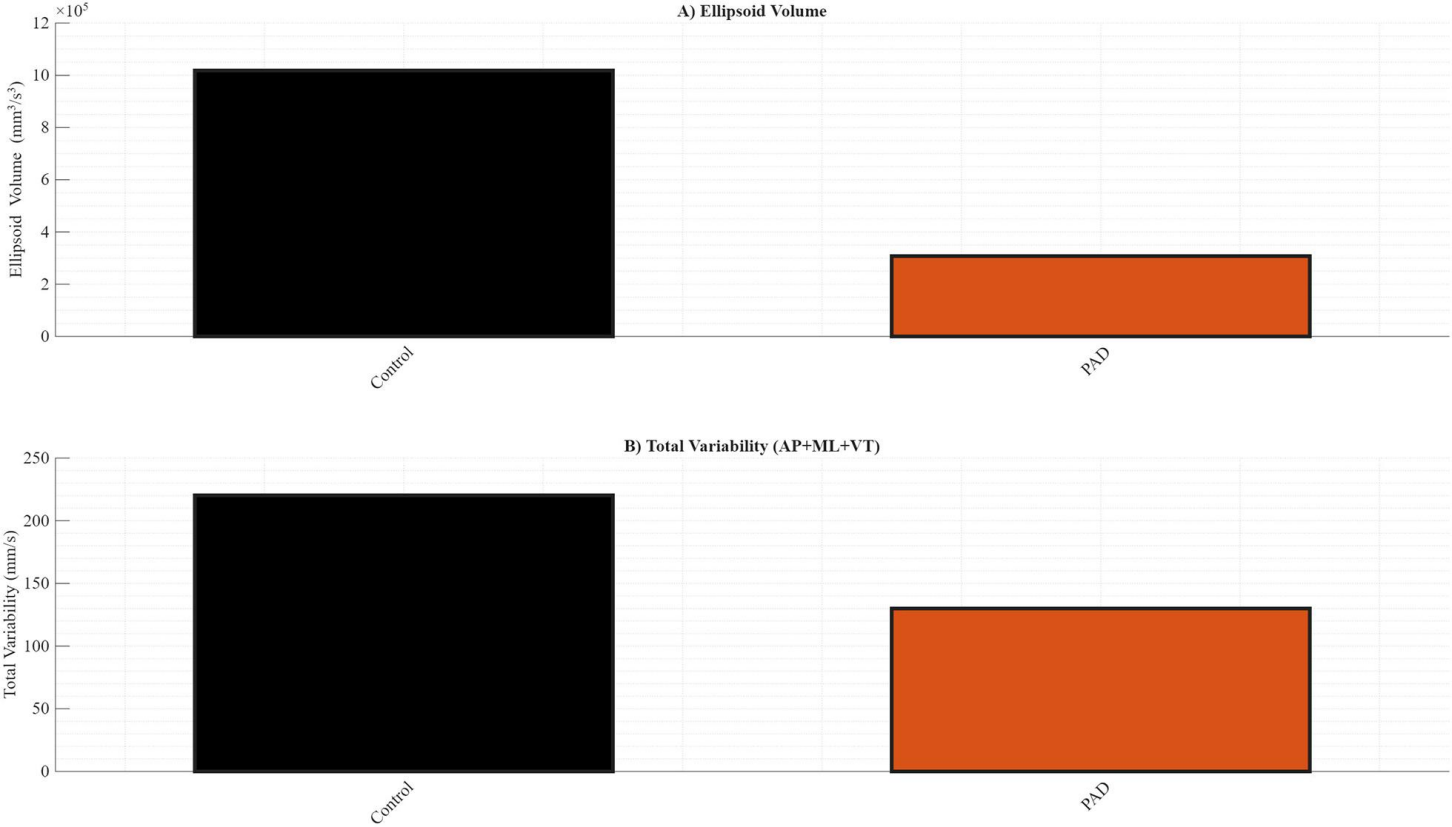
Summary of variability metrics and correlations across walking conditions for each group. **(A)** Raw values of ellipsoid volume for each condition. **(B)** Raw values of total variability for each condition.

Figure 4 provides additional insights into variability metrics and inter-directional coordination of gait stability. In Figure 4A, raw values indicate that individuals with PAD showed lower ellipsoid volume and reduced total linear variability compared to the Control group. Figure 4B shows Pearson correlation coefficients between ellipsoid volume and total variability (AP + ML + VT). While the Control group demonstrated a strong positive correlation (*r* = 0.55, *p* < 0.001), this relationship was not present and non-significant in PAD (*r* = 0.06, *p* = 0.6587), suggesting a disruption in the coordinated regulation of multidirectional stability. These findings reveal that ellipsoid volume was significantly lower in PAD (*p* < 0.001), and VT variability was also significantly reduced (*p* < 0.001). In contrast, no significant differences were observed for AP (*p* = 0.1062) or ML (*p* = 0.6467) variabilities. The diminished correlation in the PAD group reinforces the notion of impaired integration of directional control strategies, consistent with a disrupted dynamic stability profile.

Within-group correlation analysis revealed a moderate positive association between ellipsoid volume and total variability in the control group (*r* = 0.55, *p* < 0.001), whereas no significant association was observed in the PAD group (*r* = 0.06, *p* = 0.66). Fisher's *r*-to-*z* transformation demonstrated that the strength of this relationship differed significantly between groups (*z* = 2.78, *p* = 0.005), indicating altered coupling between ellipsoid volume and total variability in individuals with PAD.

## 4 Discussion

This study applied the GTS methodology to examine three-dimensional, phase-dependent gait stability in individuals with PAD compared to age-matched healthy controls. We assessed whether PAD is associated with impaired dynamic stability across the gait cycle by using information that conventional event-based measures, such as the MoS, cannot fully capture (19). As expected, patients with PAD walked with a significantly lower mean ellipsoid volume compared to controls, indicating a globally constrained gait pattern with less room for the COM to vary. Although walking speed may contribute to differences in COM variability, the observed phase-specific patterns suggest that stability alterations in PAD are not solely attributable to reduced speed. In addition, patients with PAD showed a marked reduction in VT variability, consistent with a deliberate stiffening of vertical motion, likely due to impaired proprioceptive input or lower-limb muscle weakness (49, 50). By contrast, AP and ML variability did not differ significantly, suggesting that forward progression and side-to-side control may be preserved or masked by compensatory strategies.

### 4.1 Phase-dependent analysis

The absence of significant differences in anteroposterior and mediolateral variabilities contrasts with expectations based on discrete-event stability metrics (51). This discrepancy highlights a key methodological distinction: discrete measures such as MoS quantify instability at specific gait events (10, 15, 19), whereas the GTS framework provides a continuous, phase-resolved characterization of COM velocity behavior across the entire gait cycle (17, 18). As a result, GTS reveals preserved forward progression and lateral control during steady-state walking in individuals with PAD, while simultaneously capturing reductions in overall COM variability through ellipsoid volume. These findings suggest that PAD-related gait adaptations may be subtle, phase-specific, and distributed over time, and therefore may not be fully captured by event-based stability measures alone.

A second key observation is the markedly weaker correlation between ellipsoid volume and total variability in PAD compared to the control group, which reinforces the idea that multidirectional balance control is fragmented in this population. This interpretation is consistent with established evidence that individuals with PAD exhibit impaired neuromuscular control and altered gait regulation during walking (52–55). Discrete-event stability metrics, such as margin of stability, have been shown to capture directional instability at specific gait events (10), whereas continuous, phase-resolved approaches provide complementary insight into how stability is regulated across the gait cycle (15). Similar compensatory stability strategies have been reported in other clinical populations. For example, individuals with chronic obstructive pulmonary disease (COPD) demonstrate increased mediolateral stability during walking, potentially to reduce energetic cost and enhance balance control (41, 56, 57), while individuals with multiple sclerosis exhibit altered dynamic stability patterns during gait (58). However, these prior studies relied primarily on event-based or cycle-averaged measures, limiting their ability to detect temporally localized adaptations. In contrast, the GTS framework enables identification of phase-specific coordination strategies that may be distributed across stance and swing rather than confined to discrete gait events. The significant difference in correlation strength between groups provides additional evidence of altered multidirectional coordination in PAD. In control participants, ellipsoid volume scaled proportionally with total variability, suggesting coherent integration of directional COM fluctuations. In contrast, the absence of a significant relationship in the PAD group indicates a decoupling between the magnitude of overall variability and its three-dimensional geometric structure. This disruption may reflect impaired neuromuscular coordination or compensatory control strategies associated with vascular and sensory deficits. Importantly, this conclusion is supported by a formal Fisher r-to-z comparison rather than reliance on significance vs. non-significance alone.

The present findings indicate that reductions in gait tube ellipsoid volume in individuals with PAD are largely driven by altered vertical regulation of the center of mass, with a secondary contribution from anteroposterior variability. The strong reduction in vertical variability may reflect adaptations related to diminished push-off power, altered limb loading and unloading, or impaired shock attenuation, all of which are consistent with known vascular and muscular limitations in PAD. While mediolateral variability did not independently differ between groups, its inclusion within the GTS framework remains important for characterizing the overall geometric structure and coordination of COM motion. Thus, the multidirectional nature of GTS reflects an integrated three-dimensional representation of COM behavior across the gait cycle rather than equal contributions from each movement direction.

Previous studies have reported that individuals with PAD adopt shorter step lengths and altered temporal patterns during walking, which have been interpreted as compensatory strategies to minimize discomfort associated with intermittent claudication (52–55, 59). While such spatiotemporal adjustments may reduce pain or perceived exertion, continuous GTS-based analysis suggests that these adaptations may be accompanied by a more constrained center-of-mass control strategy. Reduced COM variability across the gait cycle may increase mechanical loading demands and limit shock attenuation, potentially elevating metabolic cost and joint stress (60, 61). Similar trade-offs between step length, shock absorption, and joint loading have been documented in other locomotor contexts, where shorter steps and reduced vertical excursion are associated with increased musculoskeletal demands (60, 61). These findings underscore the importance of continuous, phase-dependent stability analysis for understanding the functional consequences of compensatory gait strategies in PAD.

The phase-dependent statistical results shown in Figure 2 highlight a key advantage of the Gait Tube Stability framework. Significant reductions in ellipsoid volume in individuals with PAD were observed across all gait phases, with particularly pronounced differences during Loading Response, Mid Stance, and Terminal Stance—phases associated with rapid weight acceptance, sustained single-limb support, and forward progression of the body over the stance limb. Differences during Pre-Swing indicate impaired unloading and transition to swing, while significant effects during Initial Swing, Mid Swing, and Terminal Swing suggest that PAD-related alterations extend into swing-phase control and preparation for initial contact. Collectively, these findings demonstrate that gait stability impairments in PAD are not confined to isolated gait events but are distributed continuously across the gait cycle. While cycle-averaged metrics summarize overall stability differences, the GTS approach reveals when, within the gait cycle, these differences are statistically meaningful and how they evolve across stance and swing phases. Importantly, the phase-dependent insights from GTS identify critical instability periods, such as during weight acceptance, that are not captured by traditional MoS analyses limited to discrete events, such as heel strike (19). This broader temporal resolution enables more targeted identification of vulnerable gait phases, as illustrated in Figure 2, and may inform phase-specific rehabilitation strategies. Compared with perturbation-based studies in static or standing conditions, in which patients with PAD showed lateral deviations in the center of gravity (8), the GTS findings from steady-state walking indicate a proactive constraint on lateral motion. This may reflect a compensatory response driven by fear of falling or increased trunk and limb stiffness (62). These results align with the work of Sutton (62), who emphasized the role of neuromuscular control adaptations in pathological gait (50). However, GTS complementary provides a dynamic, temporally resolved framework that reveals how such adaptations unfold across the gait cycle, offering insights that static or event-based methods cannot.

The apparent divergence between prior MoS findings and the present GTS results reflects fundamental differences in what these metrics quantify rather than a true contradiction. MoS is a discrete, event-based measure typically evaluated at specific gait events, such as heel strike, and captures the instantaneous mechanical margin between the extrapolated center of mass and the base of support (10, 15). Previous studies using MoS have reported significant anteroposterior and mediolateral differences in individuals with PAD, suggesting reduced instantaneous stability margins at critical gait events (24, 40). In contrast, the GTS framework provides a continuous, phase-dependent assessment of center-of-mass velocity variability and multidirectional coordination across the entire gait cycle. As such, AP and ML alterations evident at isolated events may not translate into sustained differences in continuous variability across gait phases. These findings indicate that MoS and GTS capture complementary but distinct aspects of gait stability, with MoS emphasizing instantaneous mechanical stability and GTS emphasizing temporal organization and multidirectional coordination of COM dynamics. Together, these measures provide a more comprehensive understanding of gait stability impairments in PAD.

The largest difference in ellipsoid volume between PAD patients and controls occurred during 10%–20% of the gait cycle, corresponding to weight acceptance and early mid-stance—a critical period for balance. This phase involves foot flattening, shock absorption, and forward COM progression, requiring coordinated joint actions and neuromuscular control (19). In PAD, deficits in strength, proprioception, and coordination can impair stability, increasing fall risk. Consequently, PAD patients often adopt conservative, low-variability gait patterns during this phase. These findings align with Myers et al., who reported altered motor strategies during weight transfer as a source of instability in PAD (21, 22, 24).

Prior work has reported altered LyEs in individuals with PAD, indicating increased sensitivity to small perturbations and reduced local dynamic stability (22, 24). While these findings provide important insight into neuromotor control, Lyapunov-based measures do not identify the specific gait phases during which instability develops or distinguish directional contributions to COM regulation (15, 16). In contrast, GTS complements these approaches by resolving phase-specific, multidirectional COM velocity variability (17), allowing identification of stance-related phases in which PAD-related instability is most pronounced. Together, these measures offer a more complete characterization of gait stability by capturing sensitivity to perturbations, temporal structure, and continuous phase-dependent control (17). LyEs characterize nonlinear divergence of gait trajectories in state space and provide insight into the system's susceptibility to infinitesimal disturbances (15, 16, 49). In contrast, the GTS framework does not quantify nonlinear divergence or chaos-related properties (17). Instead, GTS characterizes multidirectional COM velocity variability and coordination continuously across the gait cycle, allowing identification of phase-dependent alterations in gait stability (17). Thus, while LyE informs how gait dynamics respond to perturbations, GTS reveals when, during the gait cycle, stability-related variability and coordination are altered and how these changes are distributed across movement directions (17). Nonlinear metrics reported in the prior study, including an elevated LyE in patients with PAD (22, 24), suggest reduced gait stability. Similarly, in individuals with MS, higher LyE values have been associated with decreased dynamic stability during walking (63). However, GTS offers a new insight by capturing three-dimensional variability across all three spatial directions and the entire gait cycle. In contrast, LyE is inherently one-dimensional and often limited to a single motion component. This added dimensionality allows GTS to detect subtle compensatory patterns that may be overlooked by traditional nonlinear metrics (64, 65). While ellipsoid volume reflects overall multidirectional COM velocity dispersion, the present results demonstrate that its relationship with traditional variability metrics differs between control and PAD groups. In individuals with PAD, altered neuromuscular control and compensatory gait strategies may decouple directional variability from overall COM dispersion, thereby breaking this relationship. This finding suggests that ellipsoid volume captures distinct aspects of gait control beyond simple variability magnitude and may be particularly sensitive to pathological adaptations.

While individuals with PAD walked at slower preferred speeds than controls, the reduced gait tube volumes observed in the PAD group cannot be attributed solely to speed differences (17, 18). The Gait Tube Stability framework quantifies multidirectional and phase-dependent variability of COM velocity rather than absolute velocity magnitude (17). The persistence of significant differences during specific stance-related phases, as identified by SPM, suggests altered neuromuscular control and coordination strategies beyond a simple scaling effect of slower walking speed (17). These findings indicate that PAD-related gait adaptations involve changes in how COM motion is regulated across the gait cycle, not merely reductions in walking speed (17).

GTS offers direction-specific detail during key gait phases (Figure 3), enhancing clinical relevance. This approach aligns with postural control findings in older adults, emphasizing adaptability and complexity in maintaining dynamic balance (66). Given its sensitivity to phase-specific changes, GTS is well-suited for longitudinal monitoring of gait function, surpassing the MoS, which requires matched walking speeds for valid comparisons (67), and traditional nonlinear measures, which are often affected by preprocessing and methodological constraints (64, 65). Future work should explore integrating GTS into fall interventions such as variable-speed treadmill training (67) and perturbation-based balance exercises (64, 65). Dynamic perturbation protocols to evaluate reactive control strategies have recently been recommended as part of comprehensive gait functional stability assessments and interventions (67). GTS provides phase-specific insights that enable clinicians to pinpoint periods of reduced stability in individuals with PAD, thereby enabling targeted interventions such as phase-tailored therapy or the strategic use of assistive devices. Its temporal precision optimizes robotic and exoskeletal assistance, ensuring support is delivered during instability-prone phases such as mid-stance or push-off (17). Together, these applications highlight GTS's potential to advance personalized rehabilitation and guide the development of next-generation assistive technologies.

### 4.2 Limitations

Although the surrogate-based optimization methods demonstrated promising performance, several limitations should be acknowledged. First, based on simulations run on a standard laptop, the warranting replication in larger cohorts. Additionally, the lack of perturbation data prevents evaluation of reactive stability. Integrating perturbation-based protocols could offer a more complete view of postural control. The present findings illustrate why continuous, phase-dependent analysis provides complementary information to discrete MoS measures in PAD. Significant reductions in COM variability were observed during Loading Response, Mid Stance, and Pre-Swing—phases associated with limb loading, single-limb support, and propulsion—despite discrete stability metrics capturing only limited aspects of these dynamics. These results suggest that PAD-related gait instability is not confined to discrete gait events but reflects altered control strategies that evolve throughout stance. By quantifying multidirectional COM variability across the entire gait cycle, GTS identifies phase-specific instability patterns that may be missed by simpler event-based measures. While GTS is introduced here as a novel and sensitive measure, it was not directly compared with established metrics such as MoS, LyE, or step-time variability (32), limiting its clinical utility in this context. Comparative analysis could further validate GTS as a complementary or additional method. Finally, future studies should consider integrating GTS with artificial intelligence models to predict stability throughout the gait cycle, improving real-time assessment, adaptive device control, and early fall risk detection in clinical populations (41, 68). Because gait tube stability metrics are derived from center-of-mass velocity, walking speed is an important factor influencing their magnitude. In this study, walking speed was not experimentally controlled, as participants walked at their preferred speed to preserve ecological validity. Consequently, the constrained gait patterns observed in individuals with PAD likely arise from a combination of slower walking speed and altered neuromuscular control. However, the phase-dependent differences identified using GTS—particularly during Loading Response, Mid Stance, and Pre-Swing—suggest that PAD-related adaptations extend beyond uniform speed effects and reflect phase-specific control strategies during stance and propulsion. Future studies should examine GTS under speed-matched conditions to further disentangle the independent effects of walking speed and disease-related stability mechanisms. In addition, the GTS framework characterizes center-of-mass velocity variability and coordination but does not directly assess joint-level kinematics, muscle activation patterns, or reactive responses to external perturbations. As a result, the neuromuscular mechanisms underlying the observed alterations in stability cannot be explicitly inferred from the present analysis. Furthermore, ellipsoid volume represents an integrated geometric measure of three-dimensional variability and should not be interpreted as indicating equal contributions from each movement direction. These factors should be considered when interpreting the physiological and clinical implications of the GTS metrics.

## 5 Conclusion

The GTS methodology revealed phase-specific gait stability deficits in PAD, marked by reduced ellipsoid volumes, restricted VT variability, and impaired direction coordination. These findings reflect reduced adaptability, likely due to compensatory responses to vascular and neuromuscular deficits, which may increase metabolic cost and fall risk. By identifying when instability occurs, GTS enables targeted rehabilitation, such as variable-speed walking and perturbation training. As a sensitive and three-dimensional tool, GTS can guide personalized interventions to help preserve mobility, independence, and quality of life in patients with PAD.

## Data Availability

The original contributions presented in the study are included in the article; further inquiries can be directed to the corresponding author.
